# Dietary Capsaicin Supplementation Mitigates Calving-Induced Stress and Enhances Antioxidant Capacity, Immune Function, and Gut Microbiota in Periparturient Dairy Cows

**DOI:** 10.3390/antiox14010028

**Published:** 2024-12-29

**Authors:** Hangfan Li, Zibin Wu, Baisheng Yu, Jinyuan Chen, Chuang Yang, Yongqing Guo, Baoli Sun

**Affiliations:** Herbivore Research Laboratory, College of Animal Science, South China Agricultural University, No. 483 Wushan Road, Guangzhou 510642, China

**Keywords:** capsaicin, periparturient dairy cows, antioxidant capacity, immune function, fecal microbiota

## Abstract

This study investigated the effects of dietary capsaicin supplementation on antioxidant capacity, immune function, and gut microbiota in periparturient dairy cows. Twenty Holstein cows with an average parity of 2.5 ± 0.76, milk production of 31.30 ± 2.39 kg, and 36.10 ± 2.38 days to calving were randomly assigned to either a control group fed a basal diet or a treatment group supplemented with 1.2 g/head/day of capsaicin. The supplementation was administered during an evaluation period spanning from 28 days before delivery to 21 days after delivery using a randomized block experimental design. Results showed that capsaicin significantly reduced milk somatic cell count and pro-inflammatory cytokines (TNF-α, IL-1β, and IL-6) while enhancing serum antioxidant enzymes (SOD, GSH-Px, and CAT) and immunoglobulin levels (IgG, IgA, and IgM). Moreover, capsaicin altered gut microbiota composition, increasing the relative abundance of beneficial genera. These findings suggest that dietary capsaicin supplementation during the transition period improves lactation performance and supports immune function, as well as alleviates oxidative stress. This study highlights the potential of capsaicin as a practical dietary strategy for enhancing productivity in dairy farming.

## 1. Introduction

The transition period, spanning 3 weeks before and after parturition, is recognized as the most critical phase in the productive life cycle of dairy cows [1]. During this time, cows face significant physiological, metabolic, and behavioral challenges that frequently result in negative energy balance (NEB) because of reduced feed intake and the heightened energy demands of fetal development and early lactation [2]. NEB induces excessive fat mobilization, elevating circulating non-esterified fatty acids (NEFA) and oxidative stress markers such as malondialdehyde (MDA) [3,4]. These metabolic alterations are often accompanied by increased levels of inflammatory cytokines, including tumor necrosis factor-α (TNF-α), interleukin-1β (IL-1β), and interleukin-6 (IL-6) [5], which together exacerbate health challenges and diminish productivity in periparturient cows [6,7].

Dietary strategies have been extensively investigated as a means to mitigate negative energy balance (NEB) and alleviate oxidative and inflammatory stress during this critical period. Among these strategies, capsaicin (CAP)—a bioactive compound derived from chili peppers—has garnered significant attention because of its potent antioxidant, anti-inflammatory, and immune-enhancing properties [8,9]. Both in vitro and in vivo studies have demonstrated capsaicin’s strong antioxidant activity, including its capacity to scavenge DPPH(1,1-diphenyl-2-picryl-hydrazyl radical, DPPH), superoxide anion, and hydroxyl radicals [10,11]. Furthermore, capsaicin has shown potential as an anti-inflammatory agent, with applications in the treatment of conditions such as arthritis and psoriasis in humans [12]. Notably, the combination of capsaicin and silibinin has been reported to effectively suppress lipopolysaccharide (LPS)-induced production of nitric oxide (NO), tumor necrosis factor-alpha (TNF-α), and interleukin-6 (IL-6) [13].

While previous studies have highlighted capsaicin’s potential for improving heat tolerance and metabolic health in livestock, its effects on periparturient dairy cows—particularly concerning immune function and gut microbiota modulation—remain insufficiently understood. Therefore, this study aims to investigate the impact of dietary capsaicin supplementation on antioxidant capacity, immune response, inflammatory markers, and gut microbiota in periparturient dairy cows. We assume that dietary capsaicin supplementation will enhance antioxidant capacity, strengthen immune responses, reduce inflammatory markers, and positively modulate gut microbiota in periparturient dairy cows compared to those not receiving capsaicin. By addressing these parameters, the study seeks to clarify the role of capsaicin as a dietary intervention to support dairy cow health and productivity during the transition period.

## 2. Materials and Methods

### 2.1. Ethics Statement

The experimental design and procedures proposed in this study have been reviewed and approved by the Experimental Animal Ethics Committee of South China Agricultural University, Guangzhou, China (approval number: 201004152). All efforts were made to minimize animal suffering.

### 2.2. Animals and Experimental Design

In this study, twenty Holstein cows were selected, exhibiting an average parity of 2.5 ± 0.76, a previous milk yield of 31.30 ± 2.39 kg/day, and a time to calving of 36.10 ± 2.38 days. The cows were randomly assigned to two groups: a control group (*n* = 10) that did not receive capsaicin supplementation, and a treatment group (*n* = 10) that was administered 1.2 g/head/day of capsaicin. Capsaicin (CAP), purchased from Lidar Corporation (Guangzhou, China), was derived from chili peppers through cleaning, soaking, and extraction processes, followed by microencapsulation using microencapsulation coating technology. The final microencapsulated product contained 2% natural capsaicin as its main active ingredient. The research employed a randomized block experimental design, with the experimental period extending from 28 days prior to parturition to 21 days post-parturition. This timeframe included a prefeeding phase lasting from 28 days to 21 days before parturition, during which cows were gradually introduced to the supplementation, and a formal feeding phase spanning from 21 days before to 21 days after parturition, where capsaicin supplementation was consistently administered. We artificially defined the day of delivery as day 0. During the trial, the cows were fed with their neck collars down for 40 min following substantial feeding, while being allowed free access to feed and water in a loose pen environment. Capsaicin was uniformly incorporated into the Total Mixed Ration (TMR) diet during the daily morning feeding to ensure that each cow received the designated daily dosage. The dietary compositions were formulated in accordance with the NRC (2001) feeding standards. Prior to parturition, the feed was formulated based on dry period requirements, and post-parturition, it was adjusted to accommodate a milk yield of 40 kg/day. The detailed composition and nutrient profiles of the diets provided to the cows are outlined in Table 1.

### 2.3. Feed Sample Analysis

The feed intake of each group of cows was systematically recorded throughout the trial. Samples were collected from both the diets and refusals, and subsequently stored at −20 °C for the analysis of feed chemical composition. To ensure consistency in the nutritional content across all phases of the trial, feed samples were routinely collected and analyzed. All samples were subjected to drying at 65 °C in a forced air oven (Model 2000; Experimental Mill, Beijing, China) for a duration of 48 h. Following the drying process, the samples were ground to pass through a 1 mm screen using a Wiley mill (standard model 4; Arthur H. Thomas Co., Philadelphia, PA, USA). The concentrations of dietary crude protein (CP) and ether extract (EE) were quantified in accordance with the methods outlined by the Association of Official Analytical Chemists (AOAC). Ash (ash content) was determined by incinerating the samples in a furnace at 550 °C for 4 h. The contents of neutral detergent fiber (NDF) and acid detergent fiber (ADF) were assessed utilizing the ANKOM A-200i fiber analyzer, following the methodology established by Van Soest et al. [15].

### 2.4. Environmental and Physiological Measurements

During the experimental period, environmental conditions within the barn were monitored three times daily (08:00, 14:00, and 20:00) at three designated points along the length of the barn. The temperature and relative humidity (RH) of the barn were measured at a height of 1.5 m using a portable temperature and humidity meter (UT333, UNI-T, Dongguan, China). The heat stress index (THI) was then calculated using the following formula:

THI = 0.81 × Td + (0.99 × Td − 14.3) × RH + 46.3

where Td refers to the dry bulb temperature and RH represents the relative humidity. These environmental measurements were essential for assessing the heat stress levels affecting the cows. The comfort threshold for cows is defined based on the temperature–humidity index (THI) as follows: comfort (THI < 68), mild discomfort (68 < THI < 72), discomfort (72 < THI < 75), alert (75 < THI < 79), danger (79 < THI < 84), and emergency (THI > 84) [16].

Additionally, rectal temperature and respiratory rate were recorded at three different time points based on the expected calving date: 21 days, 14 days, and 7 days prior to parturition. Rectal temperature was measured at two time intervals each day, 07:30–08:30 and 14:00–15:00, using a rectal thermometer (GLA-M700) inserted 12 cm into the rectum of each cow. Respiratory rate was determined by counting the number of chest and abdominal wall movements per minute using a stopwatch during the same time periods. These measurements were critical for evaluating the physiological responses of the cows to the barn environment and heat stress during the transition period.

### 2.5. Milk Samples Collection and Analysis

The milk yield of the test cows was meticulously recorded on a daily basis throughout the duration of the experiment, specifically from day 1 to day 42 post-calving. The average daily milk yield was calculated over intervals of three days. Milk samples were collected at 7, 14, and 21 days after calving and were subsequently mixed in a ratio of 4:3:3. One sample was preserved with potassium dichromate, and the composition of the milk, including milk fat, milk protein, and lactose, was analyzed using the LACTOSCAN LWA dairy analyzer in a timely manner. The second sample was stored at −20 °C for the assessment of milk urea nitrogen (MUN) and antioxidant indices, which were determined using a Burton Synergy H1 Enzyme Analyzer, employing the colorimetric method as described by Ekinci and Broderick [17]. The cows were milked using a DeLaval Rotary E300 milking machine, equipped with a DeLaval Flow Indicator FI7 electronic flow meter. Milk yield data were extracted from the Dairy Star website.

### 2.6. Blood Sample Collection and Testing

Blood samples for the determination of serum biochemical parameters were collected from all enrolled cows at three time points: 7 days prenatal (based on the expected calving date), 7 days postnatal, and 21 days postnatal. The samples were collected using procoagulant blood collection tubes (BD Vacutainer™ for trace element testing with serum clot activator). After centrifugation at 1500× *g* for 10 min (Centrifuge 5810 R-Eppendorf, Hamburg, Germany), the serum samples were stored as follows: one aliquot at −20 °C for biochemical analysis and two aliquots at −80 °C for future analyses. Serum total protein (TP), albumin (ALB), globulin (GLB), triglycerides (TG), glucose (GLU), urea (UREA), alanine transaminase (ALT), aspartate transaminase (AST), alkaline phosphatase (ALP), non-esterified fatty acids (NEFA), β-hydroxybutyric acid (BHBA), cortisol (COR), triiodothyronine (T3), thyroxine (T4), calcium (Ca), and phosphorus (P) were measured using commercial colorimetric assay kits (Nanjing Jiancheng Bioengineering Institute, Nanjing, China). Immunoglobulin levels (IgG, IgA, IgM) were measured using commercial colorimetric bovine-specific kits (Beijing Hua Ying Biotechnology Research Institute, Beijing, China). Tumor necrosis factor-alpha (TNF-α), interleukin-1 beta (IL-1β), and interleukin-6 (IL-6) concentrations were determined using bovine-specific ELISA kits (Beijing Northern Institute of Biotechnology, Beijing, China). The oxidative and antioxidant indices, including superoxide dismutase (SOD), glutathione peroxidase (GSH-Px), catalase (CAT), total antioxidant capacity (T-AOC), and malondialdehyde (MDA) content, were assessed using commercial ELISA kits from Beijing Northern Institute of Biotechnology (Beijing, China). All analyses were performed using a Beckman 5800u automated biochemical analyzer for metabolic assays, an XH6080 radioimmunoassay analyzer for insulin and glucagon levels, and a Boten Synergy H1 ELISA analyzer for the oxidative and antioxidant assays.

### 2.7. Fecal Fermentation Parameter

Fecal samples were collected at 7 days and 21 days postpartum through rectal sampling. To measure pH, 10 g of feces was mixed with 10 mL of distilled water, and the mixture was homogenized before the pH was measured immediately using a METTLER TOLEDO FE28-Standard pH meter. For the analysis of volatile fatty acids (VFAs), a 20 mL filtered sample was placed into a plastic bottle containing 3 mL of 25% metaphosphoric acid and 3 mL of 0.6% 2-ethyl butyric acid (used as an internal standard), and subsequently stored at −20 °C. The concentration of VFAs was determined using an Agilent 6890B gas chromatograph, as described by Cao et al. [18]. Ammonia nitrogen was determined using the method of Su et al. [19].

### 2.8. Microflora Analyses of Fecal

Fecal samples collected at 7 and 21 days postpartum were subjected to high-throughput sequencing to assess microbial diversity. Following the extraction of fecal DNA using the CTAB method in accordance with the manufacturer’s guidelines, the concentration and quality of the DNA samples were evaluated using a NanoDrop 2000/2000C spectrophotometer (Thermo, Waltham, MA, USA). The V3 to V4 regions of the 16S rRNA gene were amplified utilizing Pyrobest DNA polymerase (Takara, DR500A), employing primer pairs designed as 341F (5′-CCTACGGGNGGCWGCAG-3′) and 806R (5′-GGACTACHVGGGTATCTAAT-3′). PCR amplification was conducted using the Phusion^®^ High-Fidelity PCR Master Mix with a GC Buffer (New England Biolabs, Frankfurt, Germany), and the resulting PCR products underwent quality control and purification. The purified PCR products were analyzed on the Illumina Novaseq 6000 platform (Personal Ltd., Shanghai, China) for equimolar paired-end sequencing. Sequencing data were processed using QIIME (V 1.8.0) software, and valid sequences were aggregated into operational taxonomic units (OTUs) by UCLUST with a similarity threshold of 97% [20]. Axonomic classification was further refined using the Basic Local Alignment Search Tool, resulting in the identification of OTUs [21]. The alpha and beta diversity of the bacterial communities were calculated using QIIME (version 1.9.1) and the R Project Vegan package (version 2.2.1) [22]. The PICRUSt database was employed to predict the functional capabilities of the bacterial communities [23]. Linear discriminant analysis effect size (LEfSe) analysis was conducted using an online tool, with a threshold of LDA score > 3 and *p* < 0.05.

### 2.9. Statistical Analysis

The data were compiled using Microsoft Excel and analyzed employing *t*-tests in SPSS version 22.0. All data are presented as mean ± standard error of the mean (SEM), with a *p*-value of less than 0.05 considered statistically significant. The experimental results were visualized using GraphPad Prism version 8.0 software. Variations in the abundance of posterior gut microbial communities were evaluated utilizing the Linear discriminant analysis Effect Size (LEfSe) tool in conjunction with the Kruskal–Wallis rank-sum test. The stacked bar plot of the community composition was visualized in the R project ggplot2 package (version 2.2.1). Between-groups Venn analysis was performed in the R project Venn Diagram package (version 1.6.16), and UpSet plot was performed in the R project UpSet R package (version 1.3.3) to identify unique and common species or OTUs. The Chao1, Shannon, Simpson, and Pielou’s Evenness indices were calculated in QIIME (version 1.9.1). Rank abundance curves were plotted in the R project ggplot2 package (version 2.2.1). The Bray–Curtis distance matrix was calculated in the R project Vegan package (version 2.5.3). Multivariate statistical techniques, including PCoA (principal coordinates analysis) of Bray–Curtis distances were generated in the R project Vegan package (version 2.5.3) and plotted in R project ggplot2 package (version 2.2.1). Statistic analysis of Adonis (also called PERMANOVA) was calculated in the R project Vegan package (version 2.5.3).

## 3. Results

### 3.1. Environmental Temperature–Humidity Index

**Figure 1:**
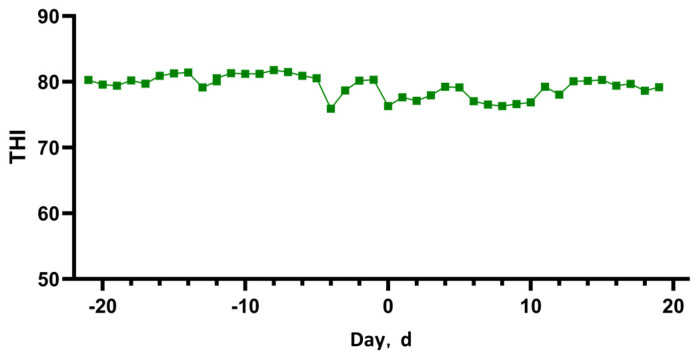
Temperature–humidity index (THI) of the cowshed during the experiment.

The daily average THI changes of the cowshed during the trial period are shown in Figure 1, with the daily average THI exceeding 72. This indicates that both groups of cows are experiencing heat stress.

### 3.2. Rectal Temperature and Respiratory Rate

**Figure 2:**
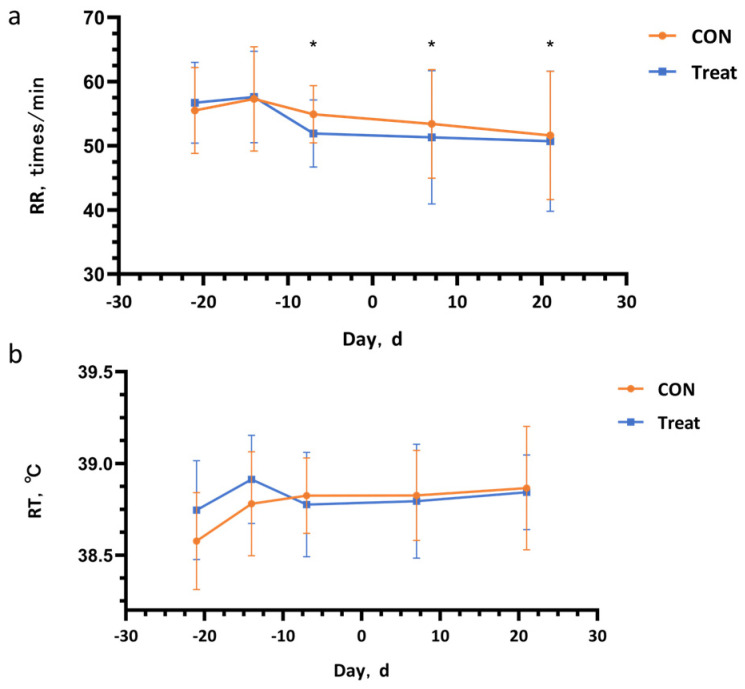
Effect of adding capsaicin to feed on the RR and RT of periparturient cows. Panel (**a**) shows the changes in rectal temperature over time, where a significant difference between the two groups is observed. Panel (**b**) describes the variation in respiratory rate, showing trends over time for both groups. * Statistical significance is indicated by asterisks (*). *p* < 0.05 denotes significant differences between groups at the same time point.

As illustrated in Figure 2, the respiratory rate (RR) of the treatment group is significantly lower than that of the control group (*p* < 0.05). However, there is no statistically significant difference in rectal temperature (RT) between the two groups (*p* > 0.05)

### 3.3. Feed Intake, Milk Yield, and Milk Composition

**Figure 3:**
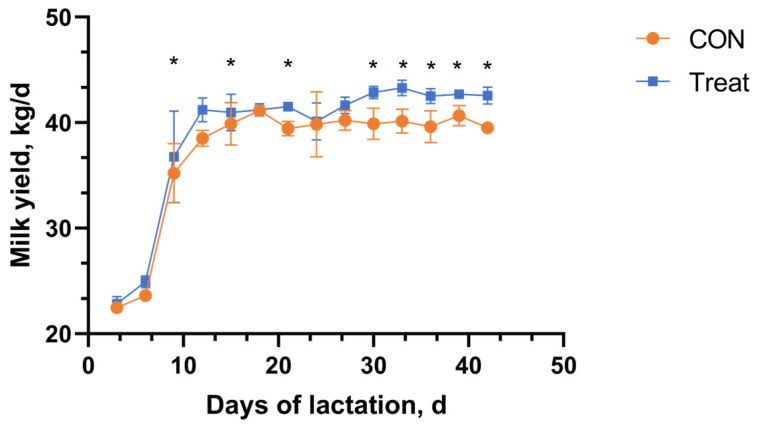
Effect of adding CAP to feed on milk production in cows. * Statistical significance is indicated by asterisks (*). *p* < 0.05 denotes significant differences between groups at the same time point.

A statistical analysis was performed on the dry matter intake (DMI) of cows from 21 days prior to parturition to 21 days postpartum, with the results detailed in Table 2. The analysis revealed no significant differences in DMI between the control (CON) and treatment (Treat) groups of cows (*p* > 0.05).·Figure 3 illustrates the changes in milk yield of dairy cows over the initial 6 weeks postpartum. During the first 2 weeks after parturition, milk production exhibited a rapid increase, which gradually decelerated starting from the third week postpartum. Cows supplemented with capsaicin demonstrated significantly higher milk yields compared to the control group at days 9, 12, 21, 30, 33, 36, 39, and 42 postpartum (*p* < 0.05). Additionally, from the fourth week postpartum onward, capsaicin supplementation was associated with an average daily increase in milk production of approximately 2.8 kg. Table 3 presents the somatic cell counts (SCCs) in the milk of dairy cows.

### 3.4. Biochemical Parameters of Blood Metabolism

As shown in Table 4, in comparison to the CON, the Treat demonstrated a statistically significant increase (*p* < 0.05) in serum total protein (TP), albumin (ALB), globulin (GLB), alanine aminotransferase (ALT), alkaline phosphatase (ALP), glucose (Glu), urea (UREA), glucagon, insulin (INS), calcium (Ca), and phosphorus (P) levels at −7 days. At the 7-day mark, the treatment group (CAP) revealed a significant reduction in serum aspartate aminotransferase (AST), ALP, non-esterified fatty acids (NEFA), and cortisol (COR) levels, while serum concentrations of Glu, triiodothyronine (T_3_), thyroxine (T_4_), and Ca significantly increased (*p* < 0.05). Additionally, at 21 days, the treatment group exhibited significantly lower concentrations of NEFA, beta-hydroxybutyrate (BHBA), glucagon, COR, T_3_, and T_4_ in bovine serum compared to the control group (*p* < 0.05).

### 3.5. Serum Immunological Parameters

The changes in the levels of serum immunological parameters are shown in Table 5. Compared to the CON, the Treat showed significant increases in the levels of IgA, IgG, and IgM in serum at −7 d, +7 d, and +21 d, while the levels of TNF-α, IL-1β, and IL-6 significantly decreased (*p* < 0.05).

### 3.6. Serum Antioxidant Parameters

Compared to the CON, the serum levels of total antioxidant capacity (T-AOC), superoxide dismutase (SOD), glutathione peroxidase (GSH-Px), and catalase (CAT) in the treatment group were significantly elevated at −7 days, +7 days, and +21 days, as shown in Table 6. Conversely, the concentration of malondialdehyde (MDA) in the serum was significantly reduced (*p* < 0.05).

### 3.7. Fecal Fermentation Parameters

Compared to the CON, the concentration of acetate in the feces of the treatment group was significantly elevated at both 7 days and 21 days (*p* < 0.05), as shown in Table 7. No significant effects were observed on other indicators *(p* > 0.05).

### 3.8. Richness, Diversity Estimates, and Fecal Bacteria Composition

#### 3.8.1. Alpha-Diversity Analysis and Beta-Diversity Analysis

**Figure 4:**
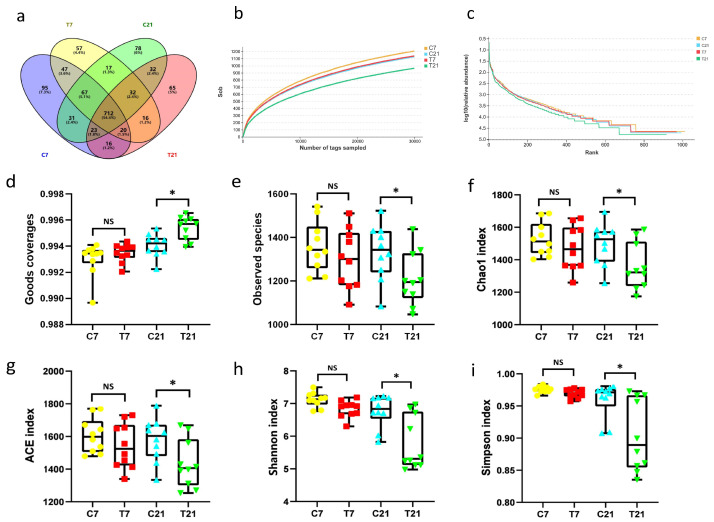
The Impact of CAP on the intestinal microbiota of periparturient cows: (**a**) Venn diagram showing shared and unique operational taxonomic units (OTUs) at 97% similarity level in the intestinal microbiota, (**b**) rarefaction curve of alpha diversity, (**c**) rank abundance curve, (**d**) goods coverage, (**e**) observed species, (**f**) Chao1 index, (**g**) ACE index, (**h**) Shannon index, (**i**) Simpson index. * *p* < 0.05, ^NS^ *p* > 0.05. C7, postpartum 7-day control group; T7, postpartum 7-day CAP group; C21, postpartum 21-day control group; T21, postpartum 21-day CAP group.

**Figure 5:**
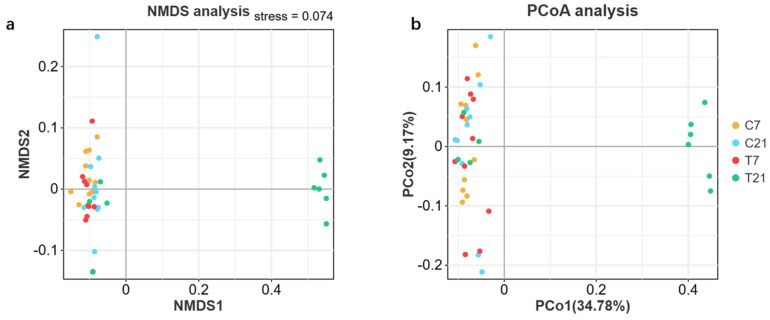
Effect of CAP on the diversity of microbiota β in fecal feces of perinatal dairy cows: (**a**) the (non-metric) multidimensional scaling (NMDS), (**b**) the principal coordinates analysis (PCoA). Each point in the graph represents a sample.

A cluster analysis was performed on 40 fecal samples utilizing a 97% similarity threshold, which led to the identification of 1308 operational taxonomic units (OTUs). The rarefaction curves for the species reached a plateau, and the Good’s coverage values for all samples exceeded 0.96, indicating that the sequencing data were appropriate for further analysis (Figure 4a–d). The individuals in the T21 and C21 groups were distinguishable from the other groups, suggesting that CAP has a significant impact on the gut microbiota of periparturient cows. We conducted β-diversity analyses using principal coordinate analysis (PCoA) and non-metric multidimensional scaling (NMDS), which revealed differences among the groups, as shown in Figure 5. Both the R value obtained from the ANOSIM analysis and the R^2^ value from the PERMANOVA analysis demonstrated similar levels of statistical significance, as shown in Table 8.

#### 3.8.2. Relative Abundance and Structure of Gut Microbiota at Phylum and Genus Levels

**Figure 6:**
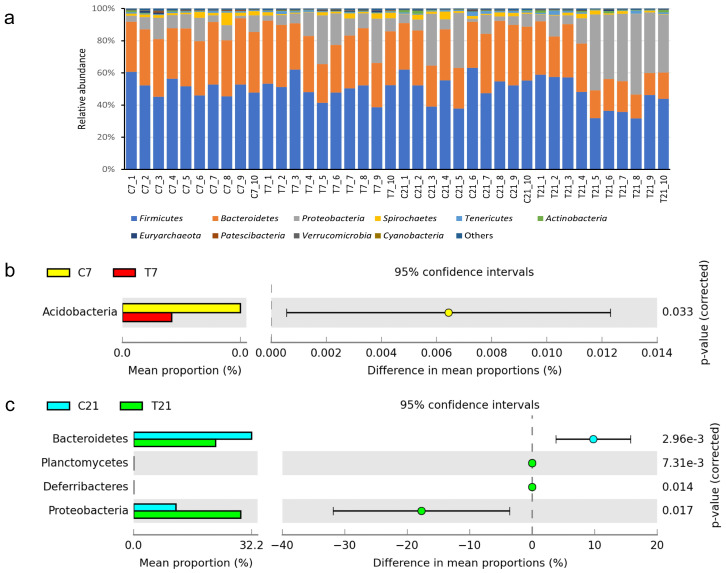
Effect of CAP on the composition of microbial flora in fecal feces of perinatal dairy cows (gate level): (**a**) relative abundance of microbiota at the phylum level, (**b**) differential microbiota map of fecal microorganisms of perinatal cows on the 7th day, (**c**) differential microbiota of perinatal dairy cows on the 21st day. T7” and “T21” refer to treatment group samples collected at 7 and 21 days postpartum, respectively. Similarly, “C7” and “C21” refer to control group samples collected at 7 and 21 days postpartum, respectively.

**Figure 7:**
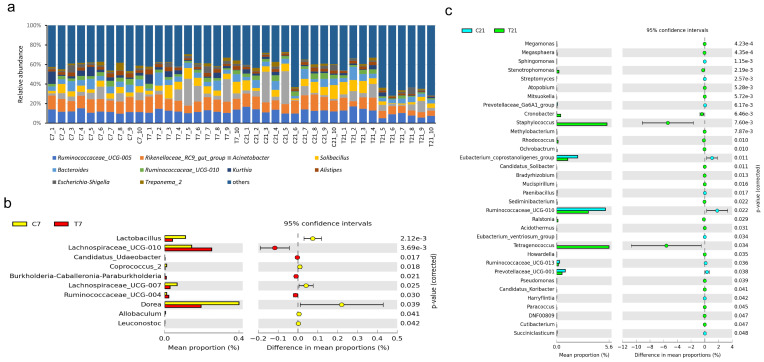
Effect of CAP on the composition of microflora in fecal feces of perinatal dairy cows (genus level): (**a**) relative abundance of microbiota at the genus level, (**b**) differential microbiota of fecal microorganisms in perinatal cows 7 days postpartum, (**c**) 21 days postpartum differential microbiota map of perinatal dairy cow microorganisms. “T7” and “T21” refer to treatment group samples collected at 7 and 21 days postpartum, respectively. Similarly, “C7” and “C21” refer to control group samples collected at 7 and 21 days postpartum, respectively.

As depicted in Figure 6 and Figure 7, the relative abundance of microbial communities in fecal samples from the posterior gut is visually represented. The 10 most abundant phyla and genera were identified, with Firmicutes, Bacteroidetes, and Proteobacteria collectively accounting for approximately 95% of the total microbiota. At the phylum level, five bacterial taxa exhibited significant differences, while at the genus level, 44 bacterial taxa demonstrated notable variations. Specifically, at 7 days postpartum, the relative abundance of four genera increased, whereas the abundance of six genera decreased. In contrast, at 21 days postpartum, the relative abundance of 23 genera increased, while that of 11 genera decreased.

## 4. Discussion

### 4.1. Environmental Temperature-Humidity Index, Rectal Temperature, and Respiratory Rate

On the basis of previous research, the temperature–humidity index (THI) is widely acknowledged as a critical metric for assessing heat stress in dairy cows [24]. A THI exceeding 72 indicates that dairy cows are subjected to heat stress. During the study period, the average THI in the experimental barn consistently remained above 72, signifying prolonged heat stress experienced by the dairy cows. Our research findings demonstrated that the administration of CAP to dairy cows 7 days prior to parturition resulted in a significant reduction in their RR. Following this intervention, the RR of the cows in the CAP group exhibited a consistent numerical decline compared to the CON group. This significant reduction in RR implies that the incorporation of CAP into the diet of periparturient dairy cows effectively alleviated heat stress. We hypothesize that this effect is likely because of the vasodilatory properties of CAP, which enhance heat exchange [25]. Capsaicin operates through the TRPV1 channel protein, which is known to induce skin vasodilation and congestion—physiological responses that are essential for blood flow and heat dissipation through sweating [26,27]. When periparturient cows are subjected to heat stress, CAP facilitates vasodilation via the TRPV1 protein, thereby promoting enhanced heat transfer, reducing RR, and mitigating heat stress.

### 4.2. DMI, Milk Production, and Milk Composition

Heat stress affects milk production, as feed intake is closely associated with milk yield [28]. The lack of significant differences in dry matter intake (DMI) between the control and capsaicin-supplemented groups is noteworthy. This result suggests that capsaicin does not negatively impact feed palatability or intake behavior in periparturient cows, which is consistent with previous studies in dairy cows [24,29]. Future studies with larger cohorts and longer observation periods are warranted to confirm these findings and explore potential dose-dependent effects. Conversely, the inclusion of CAP in the diets of periparturient cows significantly enhanced milk production on days +9, +12, +21, and +30, which may improve energy utilization efficiency and increase milk yield [30,31]. In this study, CAP supplementation was also found to significantly reduce the somatic cell count (SCC) at 7, 14, and 21 days postpartum. This reduction may not only be attributed to the inhibitory effects of CAP on the proliferation of pathogenic microorganisms responsible for mastitis but also to its anti-inflammatory properties. As shown in Table 5, CAP supplementation significantly decreased the levels of pro-inflammatory cytokines, including TNF-α, IL-1β, and IL-6, which are key mediators of inflammation. By reducing systemic and local inflammation, CAP likely improved mammary gland health, thereby contributing to the observed decrease in SCC. These results suggest that CAP may improve milk quality and mammary health in periparturient cows through a combination of inflammation modulation and potential antimicrobial effects. However, further research is needed to directly assess mammary inflammation, pathogen load, and the interplay between inflammation and lactation to elucidate the underlying mechanisms.

### 4.3. Serum, Immune Parameters, Antioxidant Indicators

Serum total protein (TP) is primarily composed of albumin (ALB) and globulin (GLB). ALB, the most abundant plasma protein, is predominantly synthesized in the liver. GLB, which is produced and secreted by plasma cells in the liver, serves as a direct indicator of the body’s immune status [32]. In the present study, the incorporation of CAP into the diet resulted in a significant increase in the levels of TP, ALB, and GLB in the serum of cows at +7 days when compared to the CON. This increase suggests that CAP may play a role in partially sustaining protein synthesis and metabolism in cows, as well as enhancing the immune function of periparturient cows.

In this study, CAP supplementation resulted in significant changes in key metabolic indicators, including blood glucose, non-esterified fatty acids (NEFA), β-hydroxybutyrate (BHBA), insulin, and glucagon levels. Specifically, CAP significantly increased blood glucose and glucagon concentrations, which were accompanied by reductions in NEFA at +7 and +21 days and BHBA at +21 days. Previous studies have shown that capsaicin can stimulate glucose absorption in the gastrointestinal tract through neural pathways, leading to elevated blood glucose levels [33]. In healthy subjects, low doses of capsaicin have been demonstrated to enhance glucose uptake while simultaneously increasing glucagon secretion [33]. Consistent with these findings, our experiment also observed an increase in blood glucose and glucagon levels in the CAP-supplemented group. This rise in blood glucose stimulates a compensatory increase in insulin secretion, as insulin is essential for facilitating glucose utilization and maintaining energy balance. However, the elevated insulin levels suppress lipolysis, thereby reducing the mobilization of body fat [34]. This suppression of fat breakdown explains the observed reductions in NEFA and BHBA concentrations. As NEFA and BHBA are metabolic byproducts of fat mobilization, their decline indicates a reduced reliance on fat catabolism for energy supply. The coordinated changes in glucose, insulin, and lipid metabolism suggest that CAP alleviates energy deficits while minimizing the risk of ketosis in periparturient cows. The results were similar to those of Oh et al. [35] These findings, although based on speculative reasoning, suggest that CAP supplementation plays a role in alleviating energy deficits and minimizing the risk of ketosis in periparturient cows. On the other hand, the insulin signaling pathway is crucial for processes such as glucose uptake, glycogen synthesis, and the synthesis of lipids and proteins [36]. During the perinatal period, vigorous fat mobilization can help address blood glucose deficiencies; however, it may also disrupt the physiological effects of insulin, leading to dysfunction in the insulin signaling pathway and an increased likelihood of insulin resistance. This condition is characterized by elevated insulin secretion, along with increased levels of free fatty acids and triglycerides. In this study, the use of CAP appears to reverse the original trend of elevated fatty acids, suggesting that CAP may have a preventive effect on insulin resistance [37]. While the observed changes in glucose, insulin, and lipid metabolism are consistent with known metabolic pathways, it is important to note that these conclusions are inferred from the data and require further validation. Specifically, the increase in blood glucose and glucagon levels, along with the subsequent reduction in NEFA and BHBA concentrations, point to a mechanism where CAP may enhance energy balance through improved glucose utilization and suppression of fat mobilization. Although these results are based on plausible metabolic pathways, they provide valuable insight into how CAP may mitigate energy negative balance in cows during the critical periparturient period. Future studies will be needed to confirm these speculative findings and establish more definitive causal relationships.

Calcium and phosphorus are vital mineral elements in the animal body, playing significant roles in various physiological and metabolic functions that are essential for growth and bone development. In adult cows, the normal blood calcium concentration is typically within the range of 2.3 ± 0.2 mmol/L, with clinical hypocalcemia being diagnosed when the blood calcium concentration falls below 1.4 mmol/L [38]. Findings from the experimental group suggest that the incorporation of CAP can elevate the blood calcium and phosphorus levels in periparturient cows to within normal ranges, thereby reducing the risk of milk fever. Research conducted by Prakash et al. [39] indicated that the administration of spicy spices, such as chili peppers, to mice can enhance the growth of intestinal villi and activate intestinal enzyme activity. Subsequent studies have demonstrated that these spicy compounds can facilitate the absorption of calcium, phosphorus, and other nutrients [40]. Therefore, it is hypothesized that CAP may alter the structural characteristics of the small intestine villi in periparturient cows, thereby promoting the absorption of calcium, phosphorus, and other nutrients, ultimately leading to an increase in blood calcium concentration.

Livestock during the production period are susceptible to inflammation both prior to and following delivery, which can significantly affect their overall health. Key components of humoral immunity, including immunoglobulins IgG, IgA, and IgM, serve as important indicators of the immune function in these animals. Additionally, certain cellular immune mediators, such as tumor necrosis factor-alpha (TNF-α) and interleukin-1 beta (IL-1β), play critical roles in the modulation of inflammatory responses [41]. The present study demonstrated that cows supplemented with CAP exhibited significantly elevated serum levels of IgG, IgA, and IgM both before and after delivery, while the serum concentrations of inflammatory mediators, including TNF-α, IL-1β, and IL-6, were markedly reduced. Capsaicin, as a TRPV1 agonist, is known to modulate cellular antioxidant defense systems through calcium-dependent signaling pathways, leading to enhanced activities of SOD, GSH-Px, and CAT. Additionally, TRPV-1 activation inhibits the NF-κB signaling pathway, a key regulator of pro-inflammatory cytokine production, thereby reducing systemic inflammation. These findings align with previous research showing that capsaicin attenuates oxidative stress and inflammation under metabolic and heat stress conditions in livestock. The phenolic groups in capsaicin further contribute to its direct antioxidant properties by scavenging peroxide radicals, thus mitigating oxidative stress induced by energy imbalances during the transition period [42,43].

During the perinatal period, the fetus experiences significant physical compression, alongside rapid fluctuations in hormone levels and metabolic status, which contribute to the production of a substantial amount of reactive oxygen species (ROS). This increase in ROS is associated with various physiological changes, including a reduction in antioxidant defense capacity and inflammation-related damage in cows, ultimately predisposing them to various diseases [44]. In the present study, we assessed alterations in oxidative stress indicators, specifically total antioxidant capacity (T-AOC), superoxide dismutase (SOD), glutathione peroxidase (GSH-Px), and catalase (CAT). Notably, SOD, CAT, and GSH-Px are integral components of the enzymatic antioxidant system, while malondialdehyde (MDA) serves as a product of lipid peroxidation under oxidative stress conditions, acting as a biomarker for oxidative stress and associated damage. T-AOC is utilized to evaluate the responsiveness of the body’s antioxidant system to free radicals and oxidative stress [45,46,47]. In this investigation, we found that the inclusion of CAP in the diet at −7 days, 7 days, and 21 days significantly enhanced the activities of T-AOC, SOD, GSH-Px, and CAT in the prepartum serum of perinatal cows, while concurrently reducing the serum levels of MDA and the concentration of ROS. CAP, characterized by its phenolic groups within its molecular structure, exhibits antioxidant properties by scavenging peroxide radicals [48]. Consequently, the administration of CAP appears to mitigate oxidative stress in perinatal cows resulting from energy imbalances.

### 4.4. Postgut Fermentation

Ruminant animals possess a diverse array of microbiota within their hindgut, where these microorganisms metabolize substrates and produce short-chain fatty acids (SCFAs), thereby forming a complex microbial ecosystem that is essential for the host’s health [49]. SCFAs have been shown to lower the overall pH of the hindgut, enhance the population of beneficial bacteria, and mitigate potential health risks [50]. In the present study, although short-chain fatty acids (SCFAs) are known to influence the overall pH of the gut, the results indicated that, aside from a significantly higher concentration of acetic acid in the feces of cows from the capsicum alkaloids group compared to the control group at both +7 and +21 days, no other significant differences were observed in the levels of various branched-chain fatty acids at different time points. Additionally, fecal pH remained stable throughout the study period. Previous research has suggested that capsicum alkaloids may modulate SCFA production by influencing the microbiota, specifically by increasing the abundance of acid-producing bacteria, which in turn affects hindgut health [51,52]. These findings imply that the administration of capsicum alkaloids to ruminants may regulate SCFA production, thereby elucidating the mechanisms through which capsicum alkaloids impact energy utilization and hindgut health.

Our study revealed a significant reduction in ammonia nitrogen concentration in the feces of cows within the experimental group. We hypothesize that this observed difference may be attributed to the influence of CAP on the modulation of gut villus morphology and associated enzymatic activities, as previously discussed. Additionally, the reduction in ammonia nitrogen concentration suggests a corresponding decrease in environmental impact.

### 4.5. Composition and Correlation Analysis of Intestinal Microbial Communities

The gut microbiota is integral to animal health, as it significantly influences immune and metabolic regulation [53]. In the present study, we performed sequencing and analysis of cow feces to assess gut microbial diversity, employing the Chao1 and ACE indices to measure species richness, and the Shannon and Simpson indices to evaluate species diversity [54]. Our findings revealed a notable decrease in alpha diversity in the T21 group compared to the C21 group, accompanied by significant differences in beta diversity. To further explore the effects of CAP on hindgut microbiota, we analyzed the microbial structure. At the phylum level, Firmicutes and Bacteroidetes were identified as the predominant phyla across all cows. Notably, the abundance of Acidobacteria diminished in the T21 group, while the abundances of Proteobacteria, Deferribacteres, and Verrucomicrobia exhibited significant increases. Acidobacteria, a prevalent gut microbe, has been linked to a reduction in the risk of diarrhea [55]. Deferribacteres, recognized for their anaerobic metabolic capabilities, may contribute to anti-inflammatory effects within the gut [56]. Furthermore, the increase in Verrucomicrobia may result in elevated acetic acid levels and decreased ammonia nitrogen concentrations in fecal short-chain fatty acids [57]. While previous studies in murine models have indicated that CAP reduces the abundance of Verrucomicrobia, our experiment demonstrated an increase in this phylum [58,59]. Despite the observed rise in Verrucomicrobia, no discernible disease symptoms were noted in the cows, and relevant blood parameters remained lower than those of the control group. This suggests that the impact of CAP on this bacterium may remain within a normal physiological range.

At the genus level within the T7 group, a decline in certain species of Lachnospiraceae and Dorea has been observed. It has been established that specific members of Lachnospiraceae and Dorea play a role in metabolic regulation and the induction of inflammation [60,61]. The antimicrobial properties of CAP have been the subject of extensive research, with its phenolic group potentially inhibiting bacterial growth by compromising membrane stability. In samples collected at 21 days postpartum, Dorea was capable of metabolizing mucin and inducing the release of pro-inflammatory cytokines, which provides further insight into the mechanisms by which CAP may reduce inflammatory factors in periparturient cows [62]. The abundance of *Ruminococcaceae UCG-010*, which is crucial for fiber degradation and is commonly found in the hindgut of ruminants, was diminished following CAP administration. It is hypothesized that CAP may enhance fiber digestion, resulting in a reduction of fermentation substrates and, consequently, a decreased relative abundance of this genus. However, there is limited research available on the *Eubacterium coprostanoligenes* group [63]. Yu et al.’s experiment demonstrated a significant negative correlation (*p* < 0.05) between the mRNA expression levels of ZO-1, ZO-2, and CLDN1 at the genus level and the relative abundance of the *Eubacterium coprostanoligenes* group. Additionally, the apparent digestibility of metabolizable energy (ME) was also negatively correlated with the relative abundance of the *Eubacterium coprostanoligenes* group unclassified. These findings suggest that the reduction of the *Eubacterium coprostanoligenes* group may reflect the potential effect of capsaicin (CAP) in enhancing gut barrier function [63].

## 5. Conclusions

This study illustrates that dietary supplementation with 1.2 g/day of CAP significantly (containing 2% capsaicin) enhances antioxidant capacity, modulates immune function, and improves the composition of gut microbiota in periparturient dairy cows. These findings indicate that CAP may serve as an effective dietary strategy to alleviate heat stress and metabolic challenges during the transition period. Future research should investigate the long-term effects and optimal dosages of capsaicin across various environmental conditions to maximize its practical application in commercial dairy farming. Although the study provides valuable insights, some limitations exist, including the relatively small sample size and the lack of explicit analysis of the interaction between treatment and time points.

## Figures and Tables

**Table 1:** Composition and nutrient content in the basal diet (%, DM basis).

Item	Pre-Partum Feed	Postpartum Feed
Ingredients		
Corn silage	29.6	20.22
Oat hay	37.43	6.28
Wheat straw	3.36	-
Alfalfa haylage	-	15.45
Ground corn grain	8.93	11
Cornmeal	-	14.23
Soybean meal	13.52	12.16
Sugar beet pulp	-	3.27
rapeseed meal	2.1	-
Sunflower meal	2.09	1.04
Cottonseed	-	5.6
Almond hulls	-	1.48
Bran	1.52	0.28
Brewer’s grains	-	3.38
Palm fat powder	-	1.17
Premix ^1^	0.69	0.53
Dicalcium phosphate	0.07	0.25
Limestone powder	-	1.12
Sodium bicarbonate	-	1.3
Potassium chloride	-	0.25
Magnesium oxide	0.51	0.47
Sodium chloride	0.17	0.53
Nutritional Components		
CP	14.5	17.6
EE	2.26	5.01
ANF	26.05	17.57
NDF	44.53	30.75
Ash	5.10	5.58
NFE ^3^	33.61	41.06
NE_L_(Mcal/kg) ^2^	1.46	1.75
Ca	0.65	0.68
P	0.48	0.52

^1^ Each kilogram of premix contains VA 1,800,000 IU; VD 500,000 IU; VE 25,650 IU; Cu 2625 mg (copper sulfate); Zn 12,000 mg (zinc sulfate); Mn 7700 mg (manganese oxide); Se 89 mg (sodium selenite); I 200 mg (calcium iodate); cobalt 85 mg (cobalt sulfate). ^2^ NE_L_ is calculated, while other nutrient values are measured according to the testing requirements outlined in the AOAC (Association of Official Analytical Chemists) method. ^3^ NFE = DM − (Ash + CP + EE + NDF) [14].

**Table 2:** Effect of adding CAP to feed on the dry matter intake (DMI) of periparturient cows (kg/d).

Time	Groups		SEM	*p*-Value
	CON	Treat		
−21 d~−14 d	14.51	14.56	0.03	0.56
−14 d~−7 d	13.35	13.31	0.12	0.61
−7 d~calving	11.42	11.50	0.53	0.90
calving~7 d	7.73	7.84	0.30	0.86
7 d~14 d	13.01	13.05	0.56	0.97
14 d~21 d	16.18	16.22	0.18	0.92

**Table 3:** Effect of adding CAP to feed on the milk composition of cows.

Item	Time/d	Groups		SEM	*p*-Value
		CON	Treat		
Milk fat, %	7	4.89	5.84	0.78	0.40
	14	4.72	4.04	0.23	0.06
	21	4.28	4.09	0.25	0.59
Milk protein, %	7	4.02	3.87	0.13	0.42
	14	3.51	3.27	0.09	0.09
	21	3.23	3.30	0.12	0.67
Lactose, %	7	4.80	4.84	0.11	0.82
	14	5.07	5.16	0.05	0.20
	21	5.11	4.96	0.16	0.52
Total milk solid, %	7	15.14	15.29	0.85	0.90
	14	13.22	13.04	0.16	0.43
	21	13.13	11.86	0.62	0.17
SCC, ×10^4^/mL	7	497.60	160.10	110.07	0.04
	14	649.10	141.90	171.95	0.05
	21	369.80	130.80	77.90	0.04
MUN, mg/dL	7	8.63	10.74	0.98	0.15
	14	9.20	11.12	0.87	0.14
	21	10.66	12.77	0.88	0.11

**Table 4:** Effect of CAP on the serum metabolic indicators of peripartum cows.

Item	Time/d	Groups		SEM	*p*-Value
		Con	Treat		
TP, g/L	−7	52.79	70.34	2.34	<0.01
	7	68.09	70.44	1.67	0.34
	21	52.20	52.97	3.23	0.87
ALB, g/L	−7	27.74	31.54	1.14	0.03
	7	33.25	31.93	1.04	0.38
	21	21.95	21.72	1.31	0.90
GLB, g/L	−7	25.06	39.80	2.30	<0.01
	7	34.85	38.51	1.86	0.18
	21	30.25	31.25	2.63	0.79
TG, mmol/L	−7	0.26	0.24	0.12	0.47
	7	0.15	0.16	0.01	0.90
	21	0.11	0.12	0.01	0.30
UREA, mmol/L	−7	3.72	5.01	0.22	<0.01
	7	4.60	4.96	0.33	0.45
	21	3.12	3.56	0.29	0.29
Glu, U/L	−7	4.13	6.24	0.16	<0.01
	7	4.25	4.14	0.40	0.85
	21	2.09	2.41	0.14	0.13
ALT, U/L	−7	14.09	21.13	1.44	<0.01
	7	16.57	18.28	1.95	0.54
	21	13.84	15.67	1.31	0.34
AST, U/L	−7	51.33	63.92	5.25	0.11
	7	107.82	84.68	5.62	0.04
	21	72.62	59.59	10.72	0.40
ALP, U/L	−7	32.65	43.08	2.84	0.02
	7	49.17	40.65	2.05	0.03
	21	29.97	29.51	2.48	0.90
NEFA, mmol/L	−7	0.18	0.13	0.03	0.37
	7	1.08	0.50	0.11	<0.01
	21	0.37	0.20	0.04	0.02
BHBA, mmol/L	−7	0.59	0.62	0.07	0.73
	7	2.31	1.68	0.40	0.28
	21	1.10	0.55	0.19	0.05
INS, μIU/mL	−7	12.66	14.18	0.32	<0.01
	7	11.37	13.27	0.36	<0.01
	21	13.59	14.59	0.30	0.03
Glucagon, pg/mL	−7	84.74	107.49	2.92	<0.01
	7	74.96	96.22	2.38	<0.01
	21	77.14	69.07	0.91	<0.01
COR, ng/mL	−7	67.76	62.06	2.25	0.08
	7	64.14	57.48	1.07	<0.01
	21	65.80	46.75	2.35	<0.01
T_3_, ng/mL	−7	1.26	1.25	0.04	0.88
	7	1.10	1.15	0.01	0.04
	21	1.19	0.99	0.03	<0.01
T_4_, ng/ml	−7	65.60	65.79	0.55	0.86
	7	63.10	64.35	0.30	0.03
	21	44.08	40.25	0.61	<0.01
Ca, mmol/L	−7	1.60	2.13	0.07	<0.01
	7	2.03	2.19	0.05	0.05
	21	1.60	1.63	0.06	0.69
P, mmol/L	−7	1.64	1.99	0.08	<0.01
	7	1.63	1.87	0.11	0.14
	21	1.57	1.67	0.09	0.49

**Table 5:** Effect of CAP on the serum immunological parameters of peripartum cows.

Item	Time/d	Groups		SEM	*p*-Value
		CON	Treat		
IgA, g/L	−7	1.51	1.88	0.07	<0.01
	7	1.65	2.23	0.07	<0.01
	21	1.39	1.79	0.07	<0.01
IgG, g/L	−7	6.39	11.27	0.39	<0.01
	7	9.70	12.01	0.52	<0.01
	21	14.67	18.29	0.64	<0.01
IgM, g/L	−7	1.14	144	0.03	<0.01
	7	1.29	1.58	0.03	<0.01
	21	1.08	1.49	0.08	<0.01
TNF-α, pg/mL	−7	73.06	58.20	1.05	<0.01
	7	64.62	53.07	0.94	<0.01
	21	60.40	49.21	2.10	<0.01
IL-1β, pg/mL	−7	24.98	18.88	0.68	<0.01
	7	21.81	16.18	0.48	<0.01
	21	21.30	16.91	0.51	<0.01
IL-6, pg/mL	−7	159.62	99.70	3.29	<0.01
	7	137.84	84.09	2.22	<0.01
	21	132.56	114.54	2.35	<0.01

**Table 6:** Effect of CAP on the antioxidant indicators of serum in perinatal dairy cows.

Item	Time/d	Groups		SEM	*p*-Value
		CON	Treat		
SOD, U/mL	−7	54.58	73.28	2.22	<0.01
	7	61.25	85.63	2.20	<0.01
	21	76.07	87.56	2.16	<0.01
GSH-Px, U/mL	−7	273.41	365.12	9.48	<0.01
	7	323.03	429.87	10.29	<0.01
	21	320.74	434.18	8.72	<0.01
CAT, U/mL	−7	34.01	42.32	1.53	<0.01
	7	37.57	46.73	1.49	<0.01
	21	37.50	46.65	1.03	<0.01
T-AOC, U/mL	−7	7.91	9.62	0.15	<0.01
	7	8.63	11.54	0.29	<0.01
	21	7.26	9.54	0.35	<0.01
MDA, nmol/mL	−7	5.30	3.73	0.29	<0.01
	7	4.94	3.41	0.26	<0.01
	21	5.22	4.30	0.14	<0.01

**Table 7:** Effect of CAP on postpartum intestinal fermentation in dairy cows.

Item	Time/d	Groups		SEM	*p*-Value
		CON	Treat		
pH	7	7.12	7.22	0.07	0.46
	21	6.97	6.99	0.06	0.84
NH_3_-N, mmol/L	7	2.46	2.39	0.19	0.87
	21	2.97	2.28	0.16	0.06
Acetate, mmol/L	7	29.59	72.22	2.23	<0.01
	21	46.93	70.98	1.62	<0.01
Propionate, mmol/L	7	11.61	12.34	1.01	0.61
	21	13.54	14.12	0.93	0.66
Butyrate, mmol/L	7	10.71	10.74	1.49	0.99
	21	10.60	11.64	1.27	0.57
Isobutyrate, mmol/L	7	0.95	1.61	0.31	0.16
	21	0.90	0.93	0.07	0.76
Valerate, mmol/L	7	2.57	1.79	0.47	0.25
	21	2.58	3.02	0.26	0.25
Isovalerate, mmol/L	7	0.86	1.61	0.47	0.28
	21	0.81	0.78	0.01	0.72

**Table 8:** ANOSIM and PERMANOVA analysis.

Item	ANOSIM		PERMANOVA	
	R	*p*	R^2^	*p*
C7 vs. T7	0.060	0.132	0.060	0.051
C21 vs. T21	0.258	0.003	0.071	0.003

## Data Availability

Data are contained within the article.
